# Targeting the sulfur-containing amino acid pathway in leukemia

**DOI:** 10.1007/s00726-024-03402-9

**Published:** 2024-07-26

**Authors:** Xiaoyan Chen, Jiahui Jin, Rui Chang, Xing Yang, Na Li, Xi Zhu, Linlin Ma, Yanfei Li

**Affiliations:** 1 The College of Medical Technology, Shanghai University of Medicine and Health Sciences, Shanghai, 201318 China; 2 Shanghai University of Medicine and Health Sciences Affiliated Zhoupu Hospital, 1500 Zhouyuan Road, Pudong new area, Shanghai, 201318 China

**Keywords:** Sulfur-containing amino acids, Metabolites, Methylation, Trans-sulfuration, Leukemia

## Abstract

sulfur-containing amino acids have been reported to patriciate in gene regulation, DNA methylation, protein synthesis and other physiological or pathological processes. In recent years, metabolism-related molecules of sulfur-containing amino acids affecting the occurrence, development and treatment of tumors have been implicated in various disorders, especially in leukemia. Here, we summarize current knowledge on the sulfur-containing amino acid metabolism pathway in leukemia and examine ongoing efforts to target this pathway, including treatment strategies targeting (a) sulfur-containing amino acids, (b) metabolites of sulfur-containing amino acids, and (c) enzymes and cofactors related to sulfur-containing amino acid metabolism in leukemia. Future leukemia therapy will likely involve innovative strategies targeting the sulfur-containing amino acid metabolism pathway.

## Introduction

**Fig. 1:**
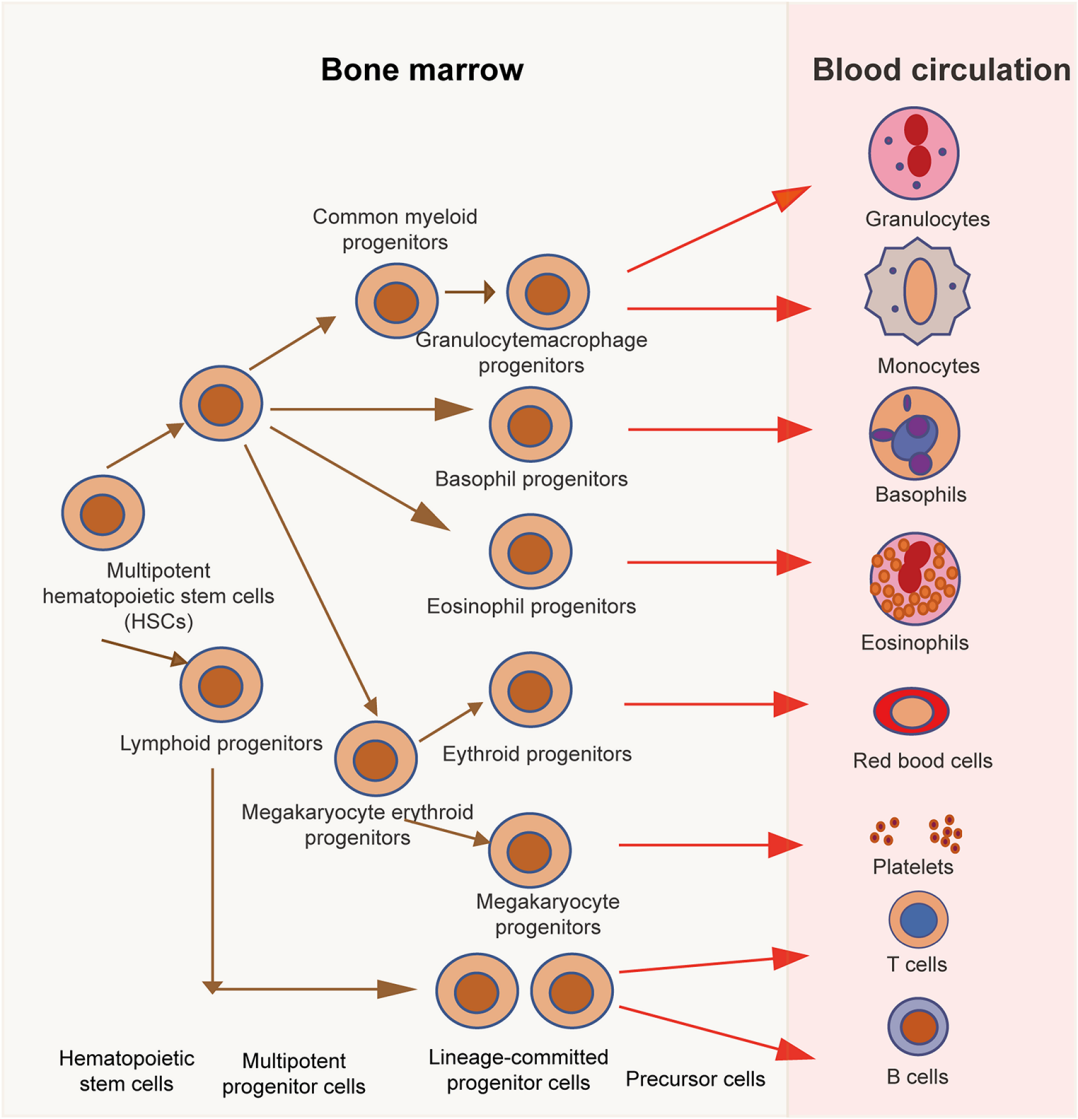
Process of hematopoietic stem cell differentiation. The figure illustrates the differentiation of hematopoietic stem cells from multipotent progenitor cells through committed progenitor cells and precursor cells to mature blood cells

Hematopoiesis is the process that involves the development and maturation of all types of hematopoietic cells. All blood cell lineages originate from hematopoietic stem cells. Based on the morphological and functional characteristics of hematopoietic cells, the hematopoietic process is typically divided into three stages: hematopoietic stem cells, lineage-committed progenitor cells, and morphologically recognizable precursor cells. Progenitor cells committed to the erythroid lineage are termed erythroid progenitor cells, while those committed to the granulocyte-monocyte lineage are called colony-forming unit-granulocyte-macrophage (CFU-GM), megakaryocytic progenitor cells are termed colony-forming units-megakaryocyte (CFU-MK), and progenitor cells of the lymphoid lineage are known as lymphoid progenitor cells. Morphologically identifiable immature cells of various lineages, exhibiting specific morphological characteristics, are termed precursor cells. These precursor cells undergo further differentiation and maturation into distinct types of terminally differentiated blood cells with specialized functions, which are subsequently released into the circulation (Fig. 1).

Leukemia is a type of malignant clonal disorder characterized by disrupted hematopoietic homeostasis, abnormal differentiation and the excessive accumulation of leukemic blasts (Culp-Hill et al. 2021; Kuek et al. 2021). The production of blood cell is inhibited by these leukemic blasts in the bone marrow and other related organs (Culp-Hill et al. 2021; Kuek et al. 2021). Numerous studies have demonstrated that amino acids metabolism plays a critical role in the onset, progression, and prognosis of leukemia. Specifically, the metabolism of sulfur-containing amino acids is intricately linked to the pathology of leukemia and its targeted therapies (Sarno et al. 2020).

### Sulfur-containing amino acids and their metabolites

**Fig. 2:**
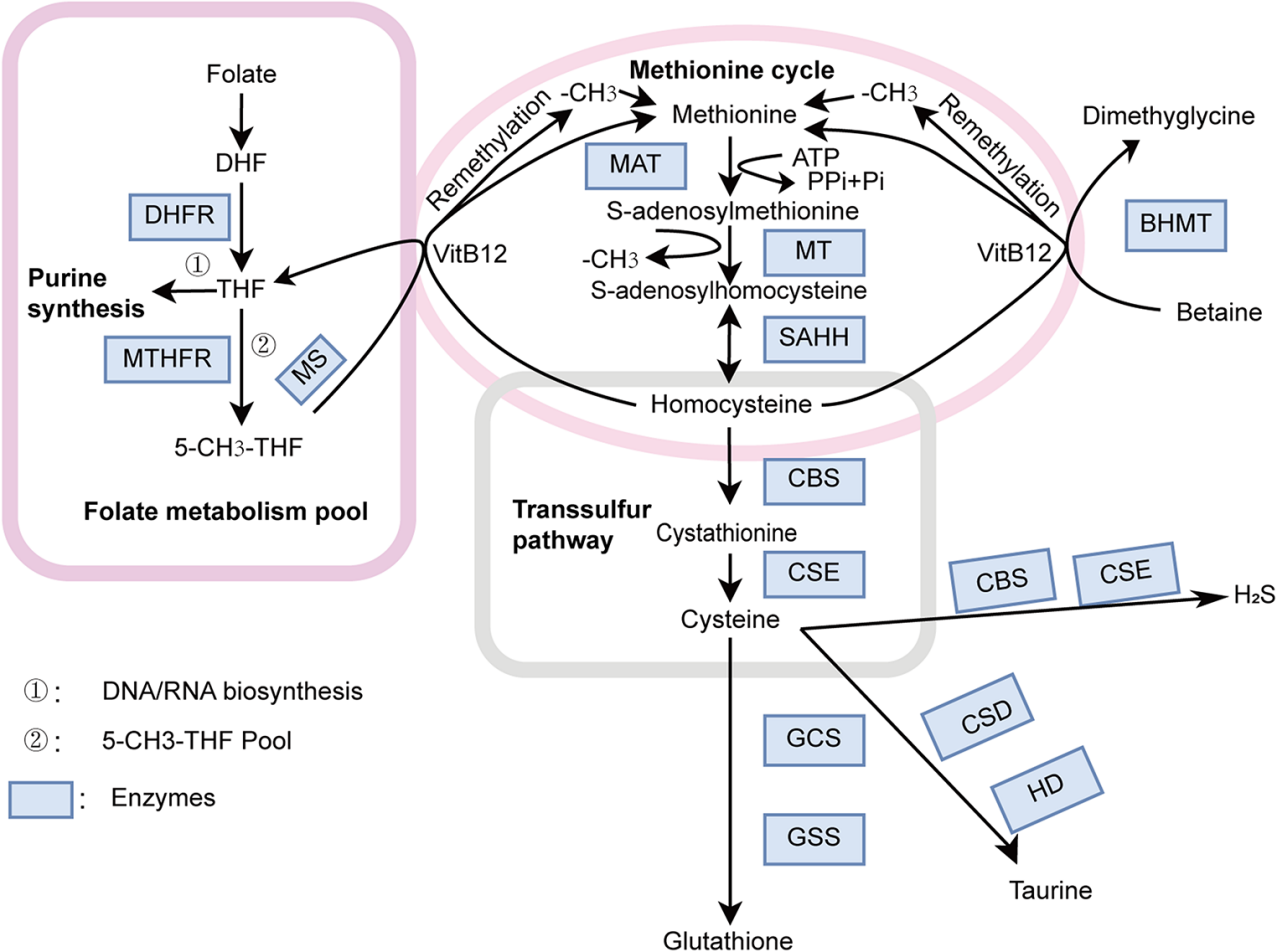
Summary of sulfur-containing amino acid metabolism. The figure explains the sulfur-containing amino acid metabolic pathway and its associated enzymes. MAT: Methionine adenosyltransferase; MT: Methyltransferase; SAHH: S-adenosylhomocysteine hydrolase; CBS: Cystathionine β-synthase; CSE: Cystathionine-γ-lyase; GCS: γ-glutamylcysteine synthase; GSS: Glutathione synthetase; MS: Methionine synthase; BHMT: Betaine homocysteine methyltransferase; CH_3_: Methyl; VitB12: vVitaminB12; THF: Tetrahydrofolate; 5-CH_3_-THF: 5-methyltetrahydrofolate; CSD: Cysteine sulfonate decarboxylase; HD: Hypotaurine dehydrogenase; H_2_S: Hydrogen sulfide; DHF: Dihydrofolate; MTHFR: Methylenetetrahydrofolate reductase; DHFR: Dihydrofolate reductase

Elemental sulfur is present in various essential bioorganic and inorganic small molecules. Sulfur can donate or accept electrons and is the atom most commonly involved in altering the oxidation state of oxygen atoms in cells. Sulfur-containing amino acids, such as methionine, homocysteine, cysteine, and taurine, are crucial molecules required by the human body (Brosnan and Brosnan 2006). Methionine, an essential amino acid, undergoes a series of metabolic steps to form homocysteine, cysteine and taurine (Stipanuk 2004). The metabolic pathway of sulfur-containing amino acids is depicted in Fig. 2.

Methionine plays a crucial role in metabolism as an active methyl donor in the human body. Methionine is converted to S-adenosylmethionine (SAM) by methionine adenosyltransferase (MAT), which is subsequently demethylated by methyltransferase (MT) to produce S-adenosylhomocysteine (Portillo et al. 2020). S-adenosylhomocysteine is then hydrolyzed to form homocysteine by S-adenosylhomocysteine hydrolase (SAHH) (Brosnan and Brosnan 2006). The conversion of homocysteine to methionine, known as remethylation, occurs primarily through two pathways. One pathway involves betaine homocysteine methyltransferase (BHMT), which utilizes a methyl group from betaine (Brosnan and Brosnan 2006; Portillo et al. 2020). Betaine is primarily derived from choline. Choline is synthesized from phosphatidylcholine, a product of phosphatidylethanolamine methylation by SAM (Blachier et al. 2020). The other pathway involves 5-methyltetrahydrofolate (5-CH3-THF) and methionine synthase (MS), with vitamin B12 (VitB12) as a coenzyme, producing tetrahydrofolate (THF) (Blachier et al. 2020). Intracellular folic acid is converted to THF from dihydrofolate (DHF) by dihydrofolate reductase (DHFR) (Oosterom et al. 2019). THF either enters the 5-CH3-THF pool for the methylation cycle via methylenetetrahydrofolate reductase (MTHFR) or is utilized in the purine cycle for DNA/RNA biosynthesis (Oosterom et al. 2019). Cystathionine-β-synthase (CBS) and cystathionine γ-lyase (CSE) are responsible for synthesizing cysteine from homocysteine in the transsulfuration pathway (Townsend et al. 2004; Wang et al. 2022). Additionally, cysteine can be used to synthesize other sulfur-containing amino acids and bioactive compounds, such as glutathione (GSH), hydrogen sulfide (H_2_S) and cysteine sulfinic acid (Blachier et al. 2020). Cysteine forms GSH through γ-glutamylcysteine synthase (GCS) and glutathione synthetase (GSS), utilizing glutamate and glycine, following the intermediate formation of γ-glutamylcysteine (Blachier et al. 2020; Wu et al. 2004). Cysteine sulfinic acid utimately produces taurine via cysteine sulfinic acid decarboxylase (CSD) and hypotaurine dehydrogenase (HD) in two steps (Blachier et al. 2020; Duszka 2022; Ramírez-Guerrero et al. 2022).

### Methionine and leukemia

The metabolism of amino acids is essential for the survival of leukemia cells. A screen for amino acid deprivation in primary leukemia stem cells and progenitor cells revealed significant dependence on methionine (Cunningham et al. 2022). The research found that methionine is primarily utilized in protein translation and for the provision of methyl groups to histones via SAM. Methionine depletion significantly impacts H3K36me3, and is accompanied by reduced RNA levels, enhanced apoptosis, and cell cycle arrest. However, methionine depletion did not affect the redox balance control in acute myeloid leukemia (AML) (Cunningham et al. 2022). Disruption of methionine/SAM metabolism either by methionine deprivation or by pharmacological inhibition of downstream pathways reduces the overall cellular methylation potential, decreases relative cell numbers, and selectively induces apoptosis in established mixed lineage leukemia (MLL)-AF4 or MLL-AF6- expressing patient blasts (Barve et al. 2019). In a leukemia mouse model, administration of L-methionine significantly increased homocysteine levels, decreased tissue plasminogen activator (tPA) levels, reversed hyperfibrinolysis, and ultimately reduced hemorrhagic symptoms in acute promyelocytic leukemia (APL) (Jácomo et al. 2012). Additionally, methionine dependence was also experimentally verified in HL-60 and Jurkat acute leukemia cells. The proliferation, cell cycle, and apoptosis of HL-60 and Jurkat cells were markedly affected by methionine restriction (He et al. 2023).

### SAM and leukemia

SAM, a universal methyl donor in human cells, is synthesized from methionine by MAT. Two distinct isoforms of MAT are encoded by the genes methionine adenosyltransferase 1 A (*MAT1A*) and methionine adenosyltransferase 2 A (*MAT2A*), with *MAT1A* expressed in the liver and *MAT2A* expressed in extrahepatic tissues (Nordgren et al. 2011) The use of the MAT2A inhibitor PF-9366 and the knockdown of MAT2A via small interfering RNA (siRNA) in a human CRISPR/Cas9-MLL-rearranged (CRISPR/Cas9-MLLr) leukemia model resulted in alterations to several cellular processes, including proliferation, viability, differentiation, apoptosis, the cell cycle, and histone methylation (Secker et al. 2020). These findings provide corroborating evidence for the hypothesis that targeted therapies against the SAM-dependent methyltransferases of the MLL protein may be a viable therapeutic approach for acute leukemia with the MLL fusion gene or MLL leukemia (Chern et al. 2020). Arsenic trioxide (As2O3, also known as ATO) was initially employed in the clinical management of APL by Chinese researchers in the 20th century (Chen and Ding 2024; Zhang et al. 2018). Subsequently, it has been established as a primary therapeutic option for APL, as indicated by numerous clinical guidelines for leukemia (Chen and Ding 2024). However, As2O3 may alter the levels of S-adenosylhomocysteine but not SAM in the treatment of APL (Khaleghian et al. 2014).

### Homocysteine and leukemia

The homocysteine-treated human acute monocytic leukemia cell line (THP-1) exhibited inhibition of autophagosome formation via the AMPK-mTOR-TFEB signaling pathway (Yang et al. 2022). Elevated plasma homocysteine levels are also used to assess the risk of toxicity associated with methotrexate (MTX), whether used alone or in combination, in acute lymphoblastic leukemia and Burkitt lymphoma (Kubota et al. 2014; Pinnix et al. 2017; Zahra et al. 2020). Furthermore, melatonin treatment has been shown to mitigate MTX toxicity, as evidenced by reduced homocysteine level (Chen et al. 2020).

### Cysteine and leukemia

Intracellular cysteine comes from two pathways, one involves conversion from intracellular methionine, and the other involves uptake via the cystine-glutamate antiporter known as system x_c_^−^ and the alanine/serine/cysteine/threonine (ASCT) neutral amino acid transporter encoded by *SLC1A4* (Cunningham et al. 2024; Liu et al. 2020; Ma et al. 2023). The ASCT transporter imports cysteine from the extracellular environment, whereas this is not the primary pathway for intracellular cysteine due to its chemical instability in the extracellular environment (Fu et al. 2019; Liu et al. 2020; Yin et al. 2016). The system x_c_^−^ cystine/glutamate exchange antiporter is a heterodimer consisting of a light chain subunit (xCT or SLC7A11) and a heavy chain subunit (CD98hc or SLC3A2), which imports cystine and exports glutamate (Liu et al. 2020). Subsequently, cystine is reduced to cysteine for the formation of GSH (Liu et al. 2020). Literature reported that cysteine cannot be synthesized from methionine in human lymphocytes and leukemia cells and are therefore highly dependent on the uptake pathway from the microenvironment (Liu et al. 2020; Ma et al. 2023). As a result, cystine import is the primary source of intracellular cysteine, particularly for hematopoietic tumor cells (Liu et al. 2020; Ma et al. 2023).

Blockade of the system x_c_^−^-GSH axis has proven remarkably effective against a wide range of tumors, including hematopoietic malignancies (Liu et al. 2020; Ma et al. 2023; Pardieu et al. 2022; Zhang et al. 2012). SLC3A2 is implicated in the glutamate dehydrogenase 1 (GDH1)-dependent progression of AML cells (Ma et al. 2023). When GDH1 is downregulated, intracellular cysteine is diminished due to disruption of the cystine-glutamate transporter resulting from decreased SLC3A2 expression. This exacerbates GSH depletion and disrupts redox homeostasis, ultimately leading to ferroptosis in AML cells (Ma et al. 2023).

Targeting key amino acid metabolic pathways through matrix-leukemia interactions is an effective means for treating residual leukemia cells (Boutter et al. 2014; Tabe et al. 2019). One such phenomenon is the dependence of chronic lymphocytic leukemia (CLL) cells on extracellular cysteine for survival. The study by Zhang et al. illustrates that patients’ CLL cells lack xCT expression, restricting the inward flow of cystine. Coincidentally, however, bone marrow stromal cells expressing high levels of xCT can efficiently utilize the cystine antiporter to intake cystine and synthesize cysteine within themselves. Cysteine is then released into the microenvironment and taken up by CLL cells leading to increased GSH synthesis, which enhances the survival of the leukemia cells and protects them from drug-induced cytotoxicity (Zhang et al. 2012). Furthermore, inhibition of SLC7A11 affects the function of AML cell lines in a cysteine-dependent manner (Pardieu et al. 2022).

Leukemia stem cells (LSCs) primarily dependent on cysteine to sustain energy metabolism for survival (Jones et al. 2019). Cysteine deficiency impairs glutathionylation of succinate dehydrogenase A (SDHA), thereby impairing the activity of the electron transport chain complex II (ETC II), inhibiting oxidative phosphorylation, reducing ATP levels, and leading to LSC death (Jones et al. 2019). These results have been confirmed by studies showing that cysteine depletion induces cell death in chronic myeloid leukemia (CML) and AML through ferroptosis, involving thioredoxin reductase 1 (TXNRD1) and glutathione peroxidase 4 (GPX4) (Cunningham et al. 2024; Liu et al. 2021). An encouraging study has developed an AML treatment using GSH-responsive cysteine polymer-based ferroptosis-inducing nanomedicine (GCFN), which acts as an efficient ferroptosis inducer and chemotherapeutic drug nanocarrier. GCFN not only depletes intracellular GSH and GSH-dependent GPX4, inducing ferroptosis in AML cells through lipid peroxidation, but also prolongs the lifespan of mice in leukemia models with minimal side effects (Yu et al. 2023). These results made it clear that cysteine has the potential to serve as a therapeutic strategy for combating leukemia.

### GSH and leukemia

GSH is a major antioxidant in cells. Multidrug-resistant (MDR) K562/adriamycin (ADM) leukemia cells have higher GSH levels and iron-regulatory protein 2 (IRP2), transferrin receptor, ferritin heavy chain 1 (FTH1), and GPX4 expression, which may enhance their antioxidantcapacity and protect K562/ADM cells from ferroptosis (Zhang et al. 2022). Another study confirmed the drug-resistant effect of GSH, showing that GSH levels are associated with MTX resistance. The authors identified two genes involved in GSH metabolism, gamma-glutamyltransferase 1 (GGT1) and thioredoxin reductase 3 (TXNRD3), as contributors to MTX resistance (Canevarolo et al. 2022).

In the study of the effect of compound 9b (2-chloro-3-ethyl-5,6,7-trimethoxy-1,4-naphthoquinone) on HL-60 leukemia cells, it was found that compound 9b induced mitochondrial DNA damage, cell cycle arrest, and ROS generation in HL-60 cells thereby resisting leukemia, with GSH playing a crucial role in the anti-leukemia mechanism of compound 9b (Li et al. 2018). In addition, elevated GSH levels were detected in the plasma of patients with primary or relapsed acute lymphoblastic leukemia (ALL) (Mahmoud et al. 2017).

AML is a highly aggressive malignancy characterized by oxidative stress resulting in abnormal levels of reduced and oxidized GSH (Abbas et al. 2023). The ratio of GSH to oxidized glutathione (GSSG) is a key indicator of cellular oxidative stress. The real-time, dynamic and highly sensitive detection of GSH/GSSG in AML cells is crucial for the clinical diagnosis and treatment of leukemia. Additionally, researchers have innovatively developed subcellular compartment-specific sensors to monitor GSH/GSSG and combined with high-resolution fluorescence microscopy to provide insights into the level of GSH/GSSG in cytoplasm, mitochondria, nucleus, and endoplasmic reticulum, revealing substantial heterogeneity in the dynamics of GSH/GSSG levels in different subcellular compartments (Abbas et al. 2023). Furthermore, drugs such as cytarabine and doxorubicin can alter GSH/GSSG levels in different subcellular compartments. The authors demonstrated, using live cell imaging, that different cellular compartments responded differently to drugs such as CB-839, parthenolide (PTL) and piperlongumine (PLM) (Abbas et al. 2023). This research will provide insights for AML treatment.

### GSS and leukemia

GSS is an important enzyme in the methionine metabolic pathway and in GSH synthesis. Cholez et al. reported that the downregulation of STAT5A, an important transcription factor, through stable transfection, siRNA, or TAT-fusion proteins, increased spontaneous cell death and Fas-promoted apoptosis in the leukemic pre-B cell lines NALM6 and Reh (Cholez et al. 2012). The targets of STAT5A might include the GSS, as that there are several binding sites in the promoter of the GSS for STAT5 (Lee et al. 2005). GSS was significantly downregulated following the decreased expression of STAT5A, leading to a decline in the protective effect against ROS, which might regulate apoptosis in NALM6 and Reh cells (Cholez et al. 2012).

In addition, GSS is involved in the mechanism of action of anti-tumor drugs that kill leukemia cells. Hamdoun et al. have shown that the anthelmintic niclosamide is more lethal to three hematopoietic tumor cell lines—CCRF-CEM, CEM/ADR5000 leukemia, and RPMI-8226 multiple myeloma—than to some solid cancer cell lines Hamdoun et al. 2017). GSS, a key molecule in the pathway by which niclosamide exerts its inhibitory effect on leukemia cell growth, may play a role in inhibiting leukemia cell growth by promoting ROS accumulation in leukemia cells ( Hamdoun et al. 2017). GSS catalyzes the biosynthesis of GSH from γ-glutamylcysteine and glycine in an ATP-dependent manner (Okada and Kimura 2022). Niclosamide, having a higher affinity for GSS due to its lower binding energy, competitively binds to GSS in leukemia cells, reducing the binding of GSS to ATP, thereby decreasing GSH synthesis and promoting ROS accumulation (Hamdoun et al. 2017).

### Taurine and leukemia

Taurine is nonproteinogenic amino acid generated from cysteine. In the present study, Zhou et al. used gas chromatography time-of-flight mass spectrometry of plasma and bone marrow supernatants to analyze the metabolic characteristics of patients with AML with maturation (AML-M2) (Zhou et al. 2020). The study showed that taurine not only had a higher plasma abundance in AML-M2 patients but also that the plasma level of taurine was positively associated with AML-M2 risk status (Zhou et al. 2020). However, the expression level of taurine transporter was negatively correlated with AML-M2 malignancy (Zhou et al. 2020).These results suggest that the plasma abundance of taurine has the potential to serve as a prognostic marker for patients with AML-M2.

In addition, patients with ALL often experience side effects during chemotherapy. The use of taurine increases white blood cell counts during chemotherapy in patients with ALL, resulting in a decrease in febrile episodes (Islambulchilar et al. 2015b). Furthermore, taurine supplementation attenuated chemotherapy-induced adverse effects, such as nausea and vomiting, and improved liver and kidney functions. Moreover, taurine significantly reduced serum malondialdehyde and superoxide dismutase levels (Islambulchilar et al. 2015a, c).

These valuable results suggest that taurine is not only a biomarker for malignant progression, but its application also has the potential to mitigate toxic side effects due to its antioxidant capacity in leukemia.

### H_2_S and leukemia

In mammals, H_2_S is primarily produced through the metabolism of sulfur-containing amino acids. CBS and CSE are two enzymes that catabolize L-cysteine to produce H_2_S, constituting the main pathway for H_2_S production in vivo (Fig. 2). Wang et al. reported that CBS and H_2_S levels were elevated in bone marrow mononuclear cells and K562 cells from pediatric CML patients. Inhibition of CBS reduced the malignancy of CML, accompanied by alterations in bone marrow mononuclear cells, apoptosis, cell-cycle progression, and migration in K562 cells and tumor xenografts. Endogenous H_2_S levels were reduced following shRNA knockdown of CBS and inhibition of CBS activity with aminooxyacetic acid (AOAA), leading to mitochondria-associated apoptosis and suppression of NF-κB-mediated gene expression (Wang et al. 2021). This study suggests that H_2_S and CBS could be potential targets for the treatment of CML.

### Folate, THF, 5-CH3-THF and leukemia

Intracellular folate levels can be categorized into two THF pools: (1) for the methylation cycle, and (2) for the purine cycle involved in DNA/RNA biosynthesis (Oosterom et al. 2019; Zarou et al. 2021). Numerous reports on hematological malignancies have demonstrated alterations in the folate metabolism pathway in leukemia cells (Köse et al. 2021; Mahmood and Emadi 2021; Metayer et al. 2016, 2023). A significant milestone in the therapeutic approach to childhood leukemia was the introduction of antifolate drugs by Sidney Farber over 70 years ago (Stine et al. 2022). MTX is a crucial antifolate used in the treatment of pediatric ALL, primarily by inhibiting the activities of several folate-associated enzymes (Winter et al. 2018; Xu et al. 2022; Yousef et al. 2019). The levels of 5-CH3-THF and THF reflect the efficacy and side effects of MTX, either alone or in combination, in the treatment of ALL, as the quantities of these folates determine overall folate levels (Oosterom et al. 2019).

### DHFR and leukemia

The expression level of DHFR in leukemia cell lines (Jurkat, Nalm-6, Reh, K562, and Molt-4) was significantly higher compared to normal hematopoietic cells. Therefore, DHFR and its gene amplification are potential targets for anti-leukemia drugs (Eyilcim et al. 2023; Gervasini et al. 2017; Kim et al. 2021; Kodidela et al. 2015; Milosevic et al. 2019; Oiwa et al. 2021; Wińska et al. 2019).

In a separate study, DHFR expression was measured in 96 children with ALL and 100 control individuals. The results indicated that DHFR mRNA expression was significantly higher in children with ALL compared to the controls (*P* < 0.001). Furthermore, DHFR mRNA levels were elevated in patients with B-cell lineage ALL compared to those with T-cell lineage ALL (*P* < 0.05). Survival analysis revealed that elevated DHFR mRNA levels were negatively correlated with overall survival in children with ALL (Organista-Nava et al. 2018).

### MTHFR and leukemia

MTHFR is a crucial enzyme influencing the pharmacokinetics of anti-leukemia drugs, such as MTX (Umerez et al. 2017). A meta-analysis including 26 studies involving 4,682 children with ALL and 7144 controls showed that the C677T polymorphism of MTHFR was associated with ALL in children, especially in Asian populations (Li et al. 2020). This conclusion was confirmed by a report by Frikha et al. (Frikha et al. 2021). Another systematic review and meta-analysis on patients with CLL found that the MTHFR polymorphism A1298C, but not C677T, might be related to the tumorigenesis of CLL under an allelic model (Raoufi et al. 2021). The authors suggested that other factors such as age, gender, ethnicity, gene-gene interactions and environmental conditions need to be considered to clarify the true association of MTHFR polymorphisms with CLL, and further experiments are necessary (Raoufi et al. 2021). Interestingly, a study suggested that the MTHFR 677T-1298 C haplotype is common in ALL and might be a valuable biomarker indicating high-dose MTX chemotherapy-related adverse effects (Frikha et al. 2022). Collectively, these studies indicate that genetic polymorphisms in MTHFR are widely associated with the occurrence and development of leukemia (Aly et al. 2014; Bănescu et al. 2015; Cwiklinska et al. 2020; Gutiérrez-Álvarez et al. 2016; Huang et al. 2015; Sazawal et al. 2014).

### VitB12 and leukemia

VitB12 is a crucial cofactor in the methionine cycle. VitB12 deficiency is a significant reversible cause of myelosuppression, megaloblastic anemia, poor growth, and increased susceptibility to infections (Kitaz et al. 2022; Konda et al. 2019; Sharma et al. 2021; Sutton and Mba 2017). Furthermore, VitB12 deficiency was significantly associated with toxic deaths in ALL (Tandon et al. 2015). Sharma et al. (Sharma et al. 2021) reported a case of a 14-month-old child with VitB12 deficiency presenting with symptoms suggestive of acute leukemia, including pancytopenia and severe anemia, and 19% reactive/atypical cells in the peripheral blood. After treatment with VitB12, the symptoms of acute leukemia improved dramatically (Sharma et al. 2021).

### BHMT and leukemia

Utilizing Tetra-primer Amplification Refractory Mutation System PCR (T-ARMS-PCR) and DNA sequencing, Bellampalli et al. (Bellampalli et al. 2017) evaluated the association of BHMT with ALL (Bellampalli et al. 2017). The results showed that although 18 nonsynonymous single nucleotide polymorphisms (nsSNPs) were deleterious, rs59109725, rs116634518, and rs138578732 in 3’-UTR altered the miRNA-binding site, and 11 copy number variations (CNVs) were present in the BHMT gene. Consequently, BHMT polymorphism resulted in alterations in free energy. Unfortunately, the association of BHMT polymorphism with both childhood and adult ALL was not significant (Bellampalli et al. 2017). The study noted that further evaluation with larger sample sizes, and exploration of the effects of other SNPs, CNVs, and miRNAs, are needed to fully understand the role of the BHMT gene in the pathogenesis of ALL (Bellampalli et al. 2017).

## Conclusion

The occurrence, progression, and therapeutic targets of leukemia are influenced by multiple factors. This review uniquely focuses on the impact of sulfur-containing amino acid metabolism on leukemia development and associated therapeutic targets.

Although there remain many uncertainties regarding the detailed mechanisms of sulfur-containing amino acids and their metabolites in leukemia, therapies targeting the metabolism of these amino acids hold significant potential for improving leukemia patient survival.

## Data Availability

No datasets were generated or analysed during the current study.

## Abbreviations

MAT: Methionine adenosyltransferase
MT: Methyltransferase
SAHH: S-adenosylhomocysteine hydrolase
CBS: Cystathionine β-synthase
CSE: Cystathionine-γ-lyase
GCS: γ-glutamylcysteine synthetase
GSS: Glutathione synthetase
MS: Methionine synthase
BHMT: Betaine homocysteine methyltransferase
CH3: Methyl
VitB12: VitaminB12
THF: Tetrahydrofolate
5-CH3-THF: 5-methyltetrahydrofolate
CSD: Cysteine sulfonate decarboxylase
HD: Hypotaurine dehydrogenase
H_2_S: Hydrogen sulfide
DHF: Dihydrofolate
MTHFR: Methylenetetrahydrofolate reductase
DHFR: Dihydrofolate reductase
SAM: S-adenosylmethionine
GSH: Glutathione
CSAD: Cysteine sulfinic acid decarboxylase
HD: Hypotaurine dehydrogenase
AML: Acute myeloid leukemia
MLL: Mixed lineage leukemia
tPA: Tissue plasminogen activator
APL: Acute promyelocytic leukemia
MAT1A: Methionine adenosyltransferase 1 A
MAT2A: Methionine adenosyltransferase 2 A
siRNA: Small interfering RNA
As2O3: Arsenic trioxide
THP-1: Acute monocytic leukemia cell line
ASCT: Alanine/serine/cysteine/threonine
GDH1: Glutamate dehydrogenase 1
CLL: Chronic lymphocytic leukemia
LSCs: Leukemia stem cells
SDHA: Succinate dehydrogenase A
ETC II: Electron transport chain complex II
TXNRD1: Thioredoxin reductase 1
GPX4: Glutathione peroxidase 4
MDR: Multidrug-resistant
ADM: Adriamycin
IRP2: Iron-regulatory protein 2
FTH1: Ferritin heavy chain 1
MTX: Methotrexate
GGT1: Gamma-glutamyltransferase 1
TXNRD3: Thioredoxin reductase 3
ROS: Reactive oxygen species
PTL: Parthenolide
PLM: Piperlongumine
AOAA: Aminooxyacetic acid
nsSNPs: Nonsynonymous single nucleotide polymorphisms
CNVs: Copy number variations
